# A cross-sectional survey on awareness of cancer risk factors, information sources and health behaviors for cancer prevention in Japan

**DOI:** 10.1038/s41598-022-18853-x

**Published:** 2022-08-26

**Authors:** Yoko Yamagiwa, Shiori Tanaka, Sarah Krull Abe, Taichi Shimazu, Manami Inoue

**Affiliations:** 1 Division of Prevention, National Cancer Center Institute for Cancer Control, 5-1-1 Tsukiji, Chuo-Ku, Tokyo, 104-0045 Japan; 2 Division of Behavioral Sciences, National Cancer Center Institute for Cancer Control, Tokyo, Japan

**Keywords:** Public health, Cancer

## Abstract

Due to recent increases in cancer burden worldwide, we investigated current awareness of cancer risk factors and the association between information sources and health behaviors for cancer prevention in Japan. A nationwide representative sample aged 20 years or older (563 men and 653 women) responded to a questionnaire as part of a population-based survey in December 2018. Tobacco smoking (55.7% of the mean attributable fraction of cancer risk overall) and cancer-causing infection (52.0%) were regarded more highly than other lifestyle factors as causes of cancer (obesity [36.6%], physical inactivity [31.9%], unbalanced diet [30.9%], and alcohol consumption [26.2%]). The association between information sources and health behaviors for cancer prevention was evaluated using a logistic regression model. The websites of public institutions, and health professionals were associated with a broad range of health behaviors including improving diet, exercise, cancer screening/health check-up, and abstinence from smoking/drinking. Among sources of print media, positive associations were observed between books and improving diet/exercise, brochures and cancer screening/health check-up, and advertisements and abstinence from smoking/drinking. A strategic health communication approach that utilizes various information sources and delivery channels is needed to inform the public about cancer prevention and to motivate risk-reducing behaviors in the population.

## Introduction

Cancer burden has been increasing worldwide^1–3^. In 2020, there were 19.3 million incident cancer cases worldwide and 10.0 million cancer deaths^4^. In Japan, the annual incidence of cancer was estimated to have reached nearly one million in 2018, and cancer became the top cause of mortality (27.3% of total deaths) in 2019^5^. Thus, effective cancer prevention and control measures are urgently needed, as are improved technologies for early detection and treatment of cancer.

The etiology of cancer has partially been elucidated, and is thought to involve both genetic factors that cause host predisposition^2^ and modifiable factors including lifestyle factors, infection and environmental factors as exposures^2^. A study conducted in 2005 in Japan estimated that 55% of cancer in men and 30% of cancer in women were preventable based on a systematic estimation of the population attributable fraction for risk factors of cancer: in men, the highest population attributable fraction was observed for tobacco smoking (30% of incidence) followed by infectious agents (23%), while in women, the highest population attributable fraction was observed for infectious agents (18%) followed by tobacco smoking (6%)^6^. Moreover, a Japanese population-based study showed that a combination of five favorable lifestyle factors (*i.e.* abstinence from smoking, moderate alcohol consumption, consuming minimal salt-preserved foods, being physically active, and having appropriate body mass index) was associated with a reduced risk of cancer overall compared to a combination of five unfavorable lifestyle factors (relative risk [RR] 0.57, 95% confidence interval [CI]: 0.45–0.72, *P* for trend = 0.0001 for men; RR 0.63, 95% CI 0.39–1.01, *P* for trend = 0.0003 for women)^7^.

However, a survey conducted in 2003 on awareness of cancer risk factors in the Japanese population showed that men and women thought that improving lifestyle could prevent 34% and 37% of cancer, respectively^8^. Thus, Japanese men appear to think cancer is less preventable than empirical estimates suggest, which may indicate insufficient awareness of the importance of lifestyle factors in cancer prevention and the need to raise such awareness.

According to ecological models, health behaviors can be modulated through multiple levels of factors including those at the individual, interpersonal, community, and policy levels. At the individual level in the health belief model, knowledge can modify a person’s belief of matters such as susceptibility, seriousness, benefits and barriers to a behavior, cues to action, and self-efficacy, which can induce behavior. In social cognitive theory, behavior can be determined reciprocally by personal cognitive factors, socioenvironmental factors, and supporting behavior factors. Knowledge of the health risks and benefits is a precondition for change, and information is needed to perform behavior in personal cognitive factors. According to theory, combined knowledge of a behavior’s significance and the components and skills required to perform the behavior, known as “behavioral capability,” is needed to perform a particular behavior^9^. We previously reported recommendations for cancer prevention for Japanese adults based on the results of a systematic review of articles on risk factors of cancer in Japan, and pooled analysis of relative risk from major population-based cohort studies to assess cancer risk for Japanese^10^. The recommendations included abstaining from smoking/avoiding passive smoking, limiting alcohol consumption to within about 23 g per day if drinking, consuming a nutritionally balanced diet (maintaining salt consumption at less than 8 g for men and 7 g for women per day, sufficient intake of fruit and vegetables, avoiding too much hot foods and drinks), being physically active, being an appropriate weight, and receiving tests and medical care for hepatitis virus infection and *Helicobacter pylori* (*H. pylori*) infection. We have since disseminated these recommendations to Japanese through the National Cancer Center website and brochures^11,12^.

According to the knowledge gap hypothesis, differences in knowledge between groups of differing socioeconomic status can cause a disadvantage to those of lower socioeconomic status, which could be modulated by information channels and sources^13^. The Health Information National Trends Survey (HINTS) in 2005 demonstrated that exposure to health information on internet and in newspapers/magazines was higher in younger individuals and among those who were white and more highly educated, while those with lower educational levels and those from non-white received television news. On the other hand, more accurate skin cancer beliefs and more adherent sun protection practices were higher in older individuals and among those who were white and more highly educated. It was considered to be important to plan message for skin cancer awareness and prevention and selection of channel to send messages taking characteristics of diverse population subgroups into consideration^14^. To improve information dissemination and to obtain the knowledge needed to develop dissemination and implementation research to promote cancer prevention, we examined current awareness of cancer prevention and the association between information sources and health behaviors on cancer prevention in a nationwide population-based survey in Japan.

## Methods

### Survey and subjects

The questionnaire used in this study formed part of an omnibus survey conducted by a non-profit survey agency in December 2018, similar to our previous survey conducted in 2003^8^. These studies were not sponsored by the government. The omnibus survey is a multipurpose cross-sectional survey commissioned by research institutes and companies to conduct public opinion research, social research, scientific research, and market research, among other purposes. Subjects were selected using a stratified three-stage sampling method: 31 strata were determined by combining 12 geographical areas with 3 city-scales of study areas (metropolis, other city, and town or village; designated by Local Anatomy Law as of April 1, 2018). For the first stage, the number of primary sampling units, the basic survey units established in Census 2015, were determined to allocate 4000 samples proportionally into the 31 strata according to the population aged 20 years or older in the Basic Resident Registration in 2017; 25 samples were allocated to each one primary sampling unit. Primary sampling units in each stratum were selected by random systematic sampling. For the second stage, using a housing map, we sampled one household among every three households in each primary sampling unit. For the third stage, one subject in each sampled household was selected by quota sampling according to the distribution of sex, age and city-scales proportionally to the population aged 20 years old or older in the Basic Resident Registration in 2017 at the time of the interview. After information about the aim/overview of the survey and a schedule for an interview was mailed to potential participants, an interviewer visited each family. The information stated that responding in the interview was voluntary, and that the interviewer would obtain oral informed consent. A face-to-face interview was conducted by the trained interviewer using a structured questionnaire sheet (Supplementary File 1). This study conformed to the ethical guidelines of the Declaration of Helsinki^15^. The study protocol was approved by the Institutional Review Board of the National Cancer Center, Japan (Approval number: 2018-199).

### Questionnaire

The questionnaire examined five issues: awareness of risk factors of cancer overall, interest in cancer prevention, health behaviors for cancer prevention, information sources on cancer prevention, and genetic testing for cancer risk (Supplementary File 1). Awareness of risk factors of cancer overall was determined using questions on: (1) modifiable factors of cancer risk, selected according to international and domestic recommendations and guidelines^8^, including alcohol consumption, unbalanced diet, use of food additives and pesticide chemicals, burnt fish and meat, tobacco smoking, obesity, physical inactivity, endocrine-disrupting chemicals, air pollution, occupational exposure, cancer-causing viral and bacterial infection, and stress; (2) genetic factors as non-modifiable risk factors; and (3) the fraction of cancer preventable by improving lifestyle. In the first and second questions, participants indicated the attributable fraction of cancer risk overall by selecting from the following categories: < 5%, 5 to < 10%, 10 to < 15%, 15 to < 20%, 20 to < 25%, 25 to < 30%, 30 to < 40%, 40 to < 50%, 50 to < 60%, 60 to < 70%, 70 to < 80%, 80 to < 90%, 90 to 100%, and “I don’t know” for each factor^8^. In the third question, participants were asked to provide a percent value representing the fraction of cancer they thought was preventable by improving lifestyle. In addition to interest in cancer prevention, we also asked participants to indicate which health behaviors they adopted for cancer prevention from the following (multiple answers were possible): improving diet, exercise, abstinence from smoking, abstinence from drinking, cancer screening/health check-up, relief of stress through hobbies, and health foods/supplements. We also asked participants to indicate their sources of cancer prevention information from the following (multiple answers were possible): television, radio, print media (newspapers, books, magazines, brochures from pharmacies/hospitals, and advertisements), internet (websites of public institutions or other organizations, and social media), and interpersonal sources (instructions from health professionals, health classes, and family/friends). The questions used in this study were similar to those used in a survey conducted previously^8^. Additionally, the omnibus survey also included questions about the participants’ basic attributes such as occupation and educational status. Answers provided to the questionnaire were converted to electronic data, without personal identifiers such as name, date of birth or home address.

### Statistical analysis

All analyses were performed using PROC SURVEY procedures in SAS statistical software (ver. 9.4) with variance estimates based on Taylor series linearization methods. Weights were calculated as the number of people aged 20 years old or older in the Basic Resident Registration in 2017 in the stratum/the number of respondents in the stratum. Those who did not provide a response were not included and post-stratification of demographic variables (e.g., age, sex, etc.) to match population-level proportions was not conducted in the calculation of weights, because response rate could not be calculated and age and sex were assigned by quota sampling at the final step of subject selection. We specified the combination of geographical areas and city-scales of study areas for strata and specified primary sampling units for cluster, for the population aged 20 years or older in the Basic Resident Registration in 2017. For analysis of the attributable fraction of cancer for risk factors, the mid-value in each category was assigned for categorical variables (e.g. 35 for the 30 to < 40% category), and the mean attributable fraction was calculated for each risk factor of cancer and compared by sex^8^. Responses of "I don't know" were excluded from the calculation of the attributable fraction. Presence of an interest in cancer prevention was defined by answers of “very interested” and “somewhat interested”. For analysis of multiple answers on information sources and health behaviors, respondents who selected each item were counted, while those who did not select the item were used as a reference in logistic regression analysis. We analyzed categorical variables using the Rao-Scott chi-squared test, and continuous variables for differences in the mean using the DIFF option in the DOMAIN statement in the SURVEYMEANS procedure. We analyzed the association between users’ characteristics and information sources by assigning 5 characteristics (age, sex, educational status, city-scale of study area, and interest in cancer prevention) as explanatory variables and 13 individual information sources used by respondents for cancer prevention as outcomes in logistic regression models (one model for each of the 13 outcomes) using the SURVEYLOGISTIC procedure. Moreover, we analyzed the association between information sources and health behaviors for cancer prevention by assigning individual information sources as explanatory variables and 5 items of individual health behaviors as outcomes, adjusting for age, sex, educational status, city-scale of study area, and interest in cancer prevention in logistic regression models (13 items of health behaviors for each of the 5 outcomes) using the SURVEYLOGISTIC procedure. The significance level of the explanatory variable was 0.00077 (0.05/65) for individual outcomes in both models using Bonferroni correction for multiple comparisons (there were 65 possible ways to combine the explanatory variables and outcomes: for information sources as outcomes, explanatory variables were 5 user characteristics (age, sex, educational status, city-scale of study area, and interest in cancer prevention) and outcomes were 13 individual information sources; for health behaviors as outcomes, explanatory variables were 13 information sources and outcomes were 5 individual health behaviors). Based on Bonferroni correction, 99.9231% confidence interval (CI) was calculated.

## Results

### Respondents’ demographic characteristics

The cooperation rate was 30.4% (1216 responses from subjects in 4000 sampled households) in this study. Of those who cooperated, 98.2% provided an answer, including “I don’t know,” to all questions, while the rest did not answer the question about the degree to which they believed cancer could be prevented by improving lifestyle Reasons for lack of responses were refusal to participate (n = 1122), absence from home in the survey period (n = 1071), change of address after sampling (n = 143), lack of knowledge about the address (n = 9), and other undetermined reasons (n = 439). However, response rates could not be calculated according to the formula determined by the American Association for Public Opinion Research due to lack detailed data on reasons for non-responses in this study^16^. The response rate did not differ by geographical area or city-scale of the study areas (Supplementary Table S1). Mean age of the respondents was 54.8 years, and 46.3% (n = 563) of respondents were men (Table 1). The mean age was statistically significantly lower and the educational status was statistically significantly higher among men than women.

**Table 1:** Demographic characteristics of respondents.

	Total	Men	Women	*P*-value
	n = 1216	n = 563	n = 653	
Age, years, mean (95% CI)	54.8 (53.8–55.9)	53.7 (52.4–55.0)	55.8 (54.5–57.1)	0.015^a^
**Educational status**^**c**^				
Middle school	92 (7.5 ± 0.8)	34 (5.9 ± 1.0)	58 (8.9 ± 1.1)	< 0.001^b^
High school	618 (50.8 ± 1.5)	257 (45.6 ± 2.2)	361 (55.3 ± 2.0)	
College or more	506 (41.6 ± 1.7)	272 (48.5 ± 2.3)	234 (35.7 ± 2.0)	
**Area**^**c,d**^				
Hokkaido and Tohoku	136 (11.5 ± 0.5)	61 (11.2 ± 1.0)	75 (11.8 ± 0.6)	0.762^b^
Kanto (Kanto and Keihin)	409 (33.8 ± 0.7)	188 (33.5 ± 1.3)	221 (34.0 ± 1.3)	
Chubu (Koshinetsu, Hokuriku and Tokai)	229 (18.2 ± 0.7)	112 (19.2 ± 1.2)	117 (17.3 ± 0.7)	
Kinki (Kinki and Hanshin)	185 (16.2 ± 0.5)	86 (16.3 ± 0.8)	99 (16.1 ± 0.9)	
Chugoku, Shikoku and Kyusyu	257 (20.3 ± 0.6)	116 (19.8 ± 1.0)	141 (20.7 ± 0.8)	
**Scale of study area**^**c**^				
Metropolis	346 (28.6 ± 0.6)	169 (30.2 ± 1.3)	177 (27.3 ± 1.1)	0.121^b^
Other cities	769 (62.6 ± 0.8)	344 (60.4 ± 1.4)	425 (64.4 ± 1.2)	
Town and Village	101 (8.8 ± 0.7)	50 (9.4 ± 1.2)	51 (8.3 ± 0.5)	

^a^Continuous variables were analyzed using weighted data for differences in means.

^b^Categorical variables were analyzed using weighted data by the Rao-Scott chi-squared test.

^c^Number (% of weighted frequency ± standard error).

^d^Sapporo, Sendai, Saitama, Chiba, Tokyo, Yokohama, Kawasaki, Sagami, Niigata, Shizuoka, Hamamatsu, Nagoya, Kyoto, Osaka, Sakai, Kobe, Hiroshima, Okayama, Kitakyushu, Fukuoka, and Kumamoto.

CI: confidence interval.

### Awareness of cancer risk factors

Although women generally indicated higher attributable fractions of cancer risk than men, the order of magnitude was similar in both sexes (Table 2). Tobacco smoking (55.7%, mean attributable fraction of cancer risk overall) and cancer-causing viral and bacterial infection (52.0%) were regarded highly as causes of cancer. In contrast, participants regarded the attributable fraction of cancer risk of other lifestyle factors to be much lower (obesity [36.6%], physical inactivity [31.9%], unbalanced diet [30.9%], and alcohol consumption [26.2%]) than that of other environmental factors (endocrine-disrupting chemicals [42.7%], air pollution [40.0%], occupational exposure [38.1%], and food additives and pesticides [33.9%]). While respondents thought the attributable fraction of cancer risk of genetic factors was high (51.7%), they thought a small fraction of cancers were preventable by improving lifestyle (34.6%). Missing data were found in calculation of the attributable fraction due to exclusion of responses of "I don't know" (4.4% to 13.6%) and no answer for the degree prevented by improving lifestyle (1.8%) (Table 2).

**Table 2:** Awareness of the attributable fraction of cancer causes.

	Total	Missing (%)	Men	Women	*P*-value^a^
**Preventable fraction of cancer by eliminating (%), mean (95% CI)**					
Tobacco smoking	55.7 (53.6–57.8)	4.4^b^	51.5 (49.1–54.0)	59.4 (56.9–61.9)	< 0.001
Cancer-causing viral and bacterial infection	52.0 (49.5–54.5)	12.7^b^	48.3 (45.2–51.4)	55.3 (52.3–58.2)	< 0.001
Stress	45.8 (43.7–48.0)	5.3^b^	40.7 (38.3–43.2)	50.2 (47.6–52.8)	< 0.001
Endocrine-disrupting chemicals	42.7 (40.5–44.9)	12.3^b^	39.7 (37.1–42.4)	45.2 (42.5–47.9)	0.0006
Air pollution	40.0 (37.8–42.2)	8.7^b^	36.4 (34.1–38.8)	43.1 (40.4–45.9)	< 0.001
Occupational exposure	38.1 (36.0–40.2)	13.6^b^	34.1 (31.7–36.5)	41.8 (39.1–44.5)	< 0.001
Obesity	36.6 (34.5–38.7)	8.1^b^	33.9 (31.4–36.3)	38.9 (36.4–41.5)	< 0.001
Food additives and pesticides	33.9 (31.8–36.0)	7.1^b^	30.4 (28.0–32.9)	36.8 (34.3–39.4)	< 0.001
Physical inactivity	31.9 (30.0–33.8)	7.4^b^	29.2 (27.0–31.3)	34.4 (32.0–36.7)	< 0.001
Unbalanced diet	30.9 (29.1–32.8)	7.6^b^	27.4 (25.0–29.7)	34.0 (31.8–36.3)	< 0.001
Alcohol drinking	26.2 (24.5–28.0)	10.3^b^	23.0 (21.0–25.1)	29.1 (26.8–31.3)	< 0.001
Burnt fish and meat	24.9 (23.0–26.8)	7.8^b^	22.5 (20.2–24.9)	27.0 (24.6–29.4)	0.0019
Fraction of cancer genetically determined (%), mean (95% CI)	51.7 (49.7–53.8)	6.1^b^	48.0 (45.4–50.6)	55.0 (52.5–57.4)	< 0.001
Fraction of cancer preventable by improving lifestyle (%), mean (95% CI)	34.6 (33.2–36.0)	1.8^c^	33.4 (31.6–35.2)	35.6 (34.0–37.2)	0.0276

^a^Continuous variables were analyzed using weighted data for differences in means.

^b^Responses of "I don't know" were excluded from the calculation of the attributable fraction.

^c^Unanswered.

CI: confidence interval.

### Interest in cancer prevention

A large proportion of respondents indicated they were interested in cancer prevention (n = 980, 80.8%), with the rate being significantly higher in women (n = 562, 86.3%) than in men (n = 418, 74.5%) (Supplementary Table S2). Respondents who indicated they were interested in cancer prevention tended to be older and marginally highly educated. The presence of interest in cancer prevention did not differ by the city-scale of study areas.

### Health behaviors for cancer prevention

The demographic characteristics (*i.e.* sex, age, educational status, and study area) of the respondents who indicated they engaged in any health behavior for cancer prevention were similar to those who indicated they were interested in cancer prevention (Supplementary Table S2). Among the individual health behaviors (Table 3), abstinence from smoking (38.4%) accounted for the highest proportion of health behaviors for cancer prevention in men, followed by improving diet (30.5%), whereas improving diet (44.6%) in women, followed by cancer screening/health check-up (40.0%). While the proportion who engaged in health behaviors tended to increase with age, the proportion who indicated they abstained from smoking and drinking tended to be high among both younger and older generations (Supplementary Table S3).

**Table 3:** Health behaviors for cancer prevention.

Health behavior for cancer prevention	Total	Men	Women	*P*-value
Number, mean (95% CI)	1.8 (1.7–2.0)	1.6 (1.5–1.8)	2.0 (1.9–2.2)	< 0.001^a^
Any health behaviors^c^	873 (71.6 ± 1.5)	380 (67.4 ± 2.1)	493 (75.3 ± 1.8)	0.0012^b^
Improving diet^c^	463 (38.1 ± 1.6)	172 (30.5 ± 2.1)	291 (44.6 ± 2.1)	< 0.001^b^
Abstinence from smoking^c^	438 (36.3 ± 1.7)	215 (38.4 ± 2.2)	223 (34.4 ± 2.2)	0.1424^b^
Cancer screening/health checkups^c^	415 (34.3 ± 1.6)	153 (27.6 ± 2.1)	262 (40.0 ± 2.0)	< 0.001^b^
Exercise^c^	319 (26.1 ± 1.3)	153 (26.8 ± 2.1)	166 (25.5 ± 1.6)	0.6008^b^
Abstinence from drinking^c^	253 (21.0 ± 1.4)	94 (17.0 ± 1.6)	159 (24.5 ± 2.0)	0.0005^b^
Relieve stress through hobbies^c^	230 (18.9 ± 1.2)	89 (15.7 ± 1.5)	141 (21.7 ± 1.5)	0.0033^b^
Health foods/supplements^c^	113 (9.2 ± 1.0)	38 (6.8 ± 1.1)	75 (11.3 ± 1.4)	0.0044b
Others^c^	8 (0.7 ± 0.2)	3 (0.5 ± 0.3)	5 (0.8 ± 0.4)	0.516^b^
Nowhere in particular^c^	338 (27.9 ± 1.5)	183 (32.6 ± 2.1)	155 (23.9 ± 1.8)	< 0.001^b^
Don’t know^c^	5 (0.4 ± 0.2)	0	5 (0.8 ± 0.4)	N/A

^a^Continuous variables were analyzed using weighted data for differences in means.

^b^Categorical variables were analyzed using weighted data by the Rao-Scott chi-squared test.

^c^Number (% of weighted frequency ± standard error).

CI: confidence interval; N/A: not applicable.

### Obtainment of information on cancer prevention

The majority of respondents indicated they obtained information on cancer prevention from any source (n = 1158, 95.2%) (Table 4). The most common source was television (n = 986, 81.2%), followed by print media (n = 754, 62.3%; including newspapers, books, magazines, brochures provided by pharmacies/hospitals, and advertisements), interpersonal sources including health professionals (n = 337, 27.7%; such as instructions from professionals and health classes) and family/friends (n = 333, 27.3%), the internet (n = 280, 23.1%; including websites of public institutions and other organizations, and social media), and radio (n = 111, 9.2%). Among the types of print media, newspapers were used by 42.6% (n = 515) of respondents, while books were used by 9.0% (n = 109).

**Table 4:** Information sources on cancer prevention.

	Total	Men	Women	*P*-value
Number of information sources, mean (95% CI)	2.8 (2.7–3.0)	2.6 (2.5–2.8)	3.0 (2.8–3.2)	< 0.001^a^
Any information sources^c^	1158 (95.2 ± 0.7)	524 (93.2 ± 1.0)	634 (97.0 ± 0.7)	< 0.001^b^
**Mass media sources**				
Television^c^	986 (81.2 ± 1.2)	420 (74.7 ± 1.8)	566 (86.8 ± 1.4)	< 0.001^b^
Radio^c^	111 (9.2 ± 0.8)	53 (9.5 ± 1.1)	58 (9.0 ± 1.1)	0.734^b^
Print^c^	754 (62.3 ± 1.8)	339 (60.7 ± 2.2)	415 (63.7 ± 2.1)	0.244^b^
Newspapers^c^	515 (42.6 ± 1.6)	238 (42.8 ± 2.1)	277 (42.4 ± 2.0)	0.899^b^
Books^c^	109 (9.0 ± 0.8)	46 (8.2 ± 1.0)	63 (9.7 ± 1.2)	0.365^b^
Magazines^c^	227 (19.0 ± 1.3)	104 (19.0 ± 1.9)	123 (19.0 ± 1.6)	0.975^b^
Handouts provided by pharmacy/hospital^c^	230 (19.1 ± 1.3)	90 (16.4 ± 1.5)	140 (21.4 ± 1.8)	0.023^b^
Advertisements^c^	218 (18.2 ± 1.4)	102 (18.7 ± 1.7)	116 (17.7 ± 1.7)	0.645^b^
Internet^c^	280 (23.1 ± 1.5)	138 (24.7 ± 2.0)	142 (21.6 ± 1.7)	0.187^b^
Website provided by public institution^c^	141 (11.7 ± 1.0)	68 (12.3 ± 1.5)	73 (11.1 ± 1.3)	0.530^b^
Website provided by others^c^	118 (9.7 ± 0.9)	70 (12.4 ± 1.5)	48 (7.4 ± 1.1)	0.005^b^
Social media^c^	80 (6.5 ± 0.8)	35 (6.2 ± 1.1)	45 (6.8 ± 1.1)	0.639^b^
**Interpersonal sources**				
Medical professionals^c^	337 (27.7 ± 1.5)	139 (25.1 ± 1.9)	198 (29.9 ± 2.0)	0.050^b^
Instructions from professionals^c^	288 (23.8 ± 1.5)	126 (22.8 ± 1.8)	162 (24.6 ± 1.9)	0.415^b^
Health classes^c^	108 (8.8 ± 0.9)	30 (5.3 ± 0.9)	78 (11.7 ± 1.3)	< 0.001^b^
Friends and acquaintances^c^	333 (27.3 ± 1.6)	115 (20.5 ± 1.9)	218 (33.2 ± 2.0)	< 0.001^b^
Others^c^	23 (1.8 ± 0.4)	12 (2.0 ± 0.6)	11 (1.7 ± 0.5)	0.678^b^
Nowhere in particular^c^	55 (4.5 ± 0.6)	37 (6.4 ± 1.0)	18 (2.8 ± 0.7)	0.001^b^
Don’t know^c^	3 (0.3 ± 0.1)	2 (0.4 ± 0.2)	1 (0.2 ± 0.2)	0.347^b^

^a^Continuous variables were analyzed using weighted data for differences in means.

^b^Categorical variables were analyzed using weighted data by the Rao-Scott chi-squared test.

^c^Number (% of weighted frequency ± standard error).

CI: confidence interval; N/A: not applicable.

### Factors associated with obtainment of information on cancer prevention

Older respondents were more likely to use radio (age [continuous], odds ratio [OR] = 1.03, 99.9231% CI by Bonferroni correction: 1.01–1.05; multivariate-adjusted model), newspapers (OR = 1.04, 99.9231% CI 1.02–1.05), while younger respondents were more likely to use social media (OR = 0.96, 99.9231% CI 0.93–0.98) (Supplementary Tables S3, S4). Further, women were more likely to use interpersonal sources compared with men: health classes (women *vs.* men, OR = 2.44, 99.9231% CI 1.19–5.00) and family/friends (OR = 1.76, 99.9231% CI 1.14–2.70). Moreover, respondents with higher levels of education tended to be more likely to use sources of print media, except advertisements, and internet sources, except social media, but not statistically significant. Preference for information sources did not differ by city-scale of the study areas.

### Information sources associated with health behaviors for cancer prevention

We investigated the association between information sources and health behaviors after adjusting for age, sex, educational status, city-scale of study area, and interest in cancer prevention (Table 5). Among print media, books were associated with improving diet (OR = 2.52, 99.9231% CI 1.18–5.39) and exercise (OR = 2.33, 99.9231% CI 1.12–4.85), and newspapers and magazines were associated with a broad range of health behaviors. Brochures provided by pharmacies/hospitals were associated with cancer screening/health check-up (OR = 2.31, 99.9231% CI 1.29–3.38), and advertisements were associated with abstinence from smoking (OR = 2.14, 99.9231% CI 1.19–3.83) and drinking (OR = 2.28, 99.9231% CI 1.30–3.98). Among the online sources, websites of public institutions were associated with a broad range of health behaviors including improving diet (OR = 2.11, 99.9231% CI 1.07–4.17), cancer screening/health check-up (OR = 2.12, 99.9231% CI 1.11–4.04), and abstinence from smoking (OR = 2.56, 99.9231% CI 1.22–5.37). Information obtained from health professionals including instructions and health classes was associated with a broad range of health behaviors including improving diet, exercise, cancer screening/health check-up, and abstinence from smoking/drinking (OR = 1.92 to 3.11, *P*-value = 0.0039 to < 0.0001). Information obtainment from family/friends was associated with abstinence from smoking (OR = 1.68, 99.9231% CI 1.03–2.73). In contrast, television and social media were not associated with an increase in any type of health behavior.

**Table 5:** Association between information sources used by respondents and health behaviors as outcomes for cancer prevention in multivariate-adjusted logistic regression models.

Health behaviors	Information sources	OR	95% CI	99.9231% CI	*P*-value
Improving diet	Television	1.30	0.91–1.85	0.70–2.41	0.1433
	Radio	1.41	0.91–2.18	0.66–3.01	0.1212
	Newspapers	1.60	1.21–2.12	0.98–2.61	0.0013
	Books	2.52	1.63–3.90	1.18–5.39	** < 0.0001**
	Magazines	1.85	1.37–2.48	1.10–3.10	** < 0.0001**
	Brochures by pharmacy/hospital	1.55	1.12–2.13	0.88–2.71	0.0082
	Advertisements	1.74	1.26–2.41	0.99–3.06	0.0009
	Website provided by public institution	2.11	1.43–3.12	1.07–4.17	**0.0002**
	Website provided by others	1.37	0.91–2.06	0.68–2.79	0.1268
	Social media	1.55	0.92–2.61	0.62–3.86	0.1028
	Instructions from professionals	1.92	1.46–2.52	1.19–3.09	** < 0.0001**
	Health classes	1.99	1.35–2.93	1.01–3.91	**0.0007**
	Friends and acquaintances	1.20	0.92–1.58	0.75–1.94	0.1796
Exercise	Television	1.11	0.78–1.58	0.60–2.05	0.569
	Radio	1.74	1.17–2.59	0.88–3.47	0.0062
	Newspapers	1.60	1.23–2.08	1.01–2.53	**0.0006**
	Books	2.33	1.53–3.55	1.12–4.85	**0.0001**
	Magazines	1.89	1.37–2.62	1.08–3.33	**0.0002**
	Brochures by pharmacy/hospital	1.54	1.12–2.13	0.88–2.70	0.0085
	Advertisements	1.77	1.27–2.46	0.99–3.15	0.0009
	Website provided by public institution	1.85	1.25–2.74	0.94–3.66	0.0022
	Website provided by others	1.04	0.68–1.60	0.49–2.21	0.8488
	Social media	1.21	0.73–2.00	0.50–2.92	0.4612
	Instructions from professionals	2.07	1.50–2.86	1.18–3.63	** < 0.0001**
	Health classes	1.99	1.45–2.73	1.14–3.45	** < 0.0001**
	Friends and acquaintances	1.48	1.06–2.07	0.83–2.66	0.0209
Cancer screening/health check-up	Television	1.29	0.90–1.85	0.69–2.42	0.1669
	Radio	1.32	0.83–2.11	0.58–3.00	0.2448
	Newspapers	1.61	1.25–2.07	1.04–2.49	**0.0003**
	Books	1.35	0.86–2.11	0.62–2.95	0.1907
	Magazines	1.67	1.19–2.33	0.93–2.98	0.0031
	Brochures by pharmacy/hospital	2.31	1.66–3.22	1.29–4.12	** < 0.0001**
	Advertisements	1.92	1.39–2.66	1.09–3.38	**0.0001**
	Website provided by public institution	2.12	1.46–3.07	1.11–4.04	**0.0001**
	Website provided by others	1.39	0.90–2.14	0.65–2.95	0.1386
	Social media	1.12	0.68–1.84	0.47–2.67	0.6672
	Instructions from professionals	3.11	2.30–4.21	1.83–5.28	** < 0.0001**
	Health classes	2.68	1.81–3.97	1.35–5.32	** < 0.0001**
	Friends and acquaintances	1.28	0.96–1.71	0.77–2.12	0.0935
Abstinence from smoking	Television	1.72	1.23–2.41	0.95–3.10	0.0020
	Radio	1.31	0.86–2.01	0.63–2.76	0.2079
	Newspapers	1.95	1.45–2.63	1.16–3.28	** < 0.0001**
	Books	1.62	1.05–2.50	0.76–3.44	0.0289
	Magazines	1.79	1.29–2.48	1.01–3.17	**0.0007**
	Brochures by pharmacy/hospital	1.92	1.38–2.68	1.08–3.43	**0.0002**
	Advertisements	2.14	1.52–3.01	1.19–3.87	** < 0.0001**
	Website provided by public institution	2.56	1.68–3.92	1.22–5.37	** < 0.0001**
	Website provided by others	1.20	0.78–1.85	0.57–2.55	0.4039
	Social media	1.04	0.57–1.88	0.37–2.92	0.8999
	Instructions from professionals	2.14	1.59–2.89	1.27–3.61	** < 0.0001**
	Health classes	1.97	1.25–3.11	0.89–4.37	0.0039
	Friends and acquaintances	1.68	1.27–2.22	1.03–2.73	**0.0003**
Abstinence from drinking	Television	1.44	0.95–2.17	0.70–2.95	0.0872
	Radio	1.74	1.06–2.86	0.73–4.15	0.0296
	Newspapers	1.76	1.22–2.53	0.93–3.32	0.0027
	Books	1.17	0.71–1.93	0.49–2.79	0.5342
	Magazines	1.48	1.04–2.10	0.80–2.72	0.0293
	Brochures by pharmacy/hospital	1.29	0.86–1.94	0.63–2.63	0.2217
	Advertisements	2.28	1.65–3.14	1.30–3.98	** < 0.0001**
	Website provided by public institution	1.76	1.15–2.69	0.84–3.68	0.0093
	Website provided by others	0.93	0.54–1.61	0.36–2.41	0.8029
	Social media	1.31	0.75–2.28	0.50–3.43	0.3361
	Instructions from professionals	2.29	1.68–3.12	1.34–3.92	** < 0.0001**
	Health classes	2.16	1.42–3.28	1.04–4.47	**0.0004**
	Friends and acquaintances	1.63	1.17–2.28	0.91–2.92	0.0045

Associations between information sources and health behaviors for cancer prevention were analyzed by assigning individual information sources as explanatory variables and 5 items of individual health behaviors as outcomes, adjusting for age, sex, educational status, city-scale of study area, and interest in cancer prevention in logistic regression models. The significance level was 0.00077 by Bonferroni correction for multiple comparisons and 99.9231% CI was calculated based on Bonferroni correction.

OR: odds ratio; CI: confidence interval.

Significant values are given in bold.

## Discussion

We conducted a cross-sectional nationwide population-based survey in Japan to analyze current awareness of cancer prevention and the association between information sources and health behaviors for cancer prevention. We found that tobacco smoking and cancer-causing viral and bacterial infection were the two highest perceived factors, both of which are major causes of cancer in Japan (16.6% of cancer incidence was explained by infections and 15.2% by active smoking in both sexes in 2015 in a recent analysis)^17^. Furthermore, reliable information sources including health professionals and the websites of public institutions were associated with a broad range of favorable health behaviors, and brochures and advertisements were associated with cancer screening/health check-up and abstinence from smoking and drinking.

The reform of various health policies to improve infection-related and lifestyle-related health issues over the past decade may explain some discrepancies between the present findings and those of previous studies. Awareness of tobacco smoking as a risk factor for cancer was higher in this study than that reported by a previous study (55.6% in 2018 *vs.* 43.0% in 2003)^8^. Smoking cessation therapy was first approved for coverage under Japan’s universal health insurance in 2006^18^, and the Health Promotion Act was reformed to regulate passive smoking in 2018^19^. Awareness was also higher for cancer-causing viral and bacterial infection in this study than in the previous study (52.0% in 2018 *vs.* 51.3% in 2003). Coverage of eradication therapy for *H. pylori* under Japan’s universal health insurance has gradually been extended over the past two decades (for ulcer in 2000, specific diseases such as idiopathic thrombocytopenic purpura in 2010, and gastritis in 2013)^20^. In addition, the government has introduced hepatitis measures to promote screening for viral hepatitis since 2002 and to subsidize antiviral therapy since 2008^21^. However, despite being listed in the Immunization Act since 2013, challenges related to vaccination for human papilloma virus (HPV) remain due to concerns about adverse effects^22^. More effective communication strategies are needed to alleviate anxiety about adverse effects and to provide information on the effectiveness of HPV vaccination for preventing cervical cancer among stakeholders.

The respondents of this study attributed higher fractions of overall cancer risk to each factor compared to those in a previous study^8^. This may be related to health concerns in the aging population. Nevertheless, the fraction of cancers considered preventable by improving lifestyle factors remained low, similar to the previous study (34.6% in 2018 *vs.* 35.5% in 2003)^8^. Such lack of awareness of an association between lifestyle improvement and cancer prevention might be due to system gaps in health policies. Since 1978, the Japanese government has promoted health measures to improve lifestyle-related diseases, including health check-ups and counseling by health professionals, and even reformed these to add metabolic syndrome in 2008^23,24^. However, the main aim of health check-ups and counseling sessions was initially to prevent cardiovascular diseases, while cancer control was established under an independent health policy framework due to the specificity of cancer screening and treatment. Thus, new strategies are needed to improve public understanding of the concepts and aims of improving lifestyle and preventing health issues including cancer. This is particularly important in Japan’s aging population, where disease patterns are changing and becoming increasingly complicated as more people live with multiple concurrent diseases, and causes of death are becoming more complex^1,2,25–28^.

Although health policies may have influenced participants’ awareness of infection and lifestyle factors for cancer risk in this study, multi-level interventions may be more effective for changing behavior, according to the ecological model of health behavior^9^. In this context, information could function to influence health behavior at multiple levels through various channels^9^.

We found that reliable sources of information such as the websites of public institutions, health professionals were associated with a broad range of favorable health behaviors. Print media sources such as books and newspapers have been suggested to contribute to in-depth knowledge at the individual level^13^. Moreover, the role of health professionals in providing social support may not just be limited to informational support but may also extend to appraisal support. We observed that information from family/friends was associated with abstinence from smoking in this study, and may function to provide social support through emotional as well as informational and appraisal support^9,29^. Interpersonal discussions are helpful for narrowing the knowledge gap by reinforcing information received from the media^13^. Meanwhile, brochures and advertisements were associated with health behaviors such as cancer screening/health check-up and abstinence from smoking and drinking in this study. The health belief model states that, at the individual level, information can act as a cue for action toward health behaviors. Informal information such as that from brochures/advertisements might form such cues for action^9^. Furthermore, a previous study reported that media campaigns were helpful for promoting abstinence from smoking and improving diet at the community level, although the effect of media campaigns was not examined in the study^30^.

In 2019, the Ministry of Internal Affairs and Communications in Japan reported that the proportion of internet users had increased even among older individuals (89.9% of total, 74.2% of those aged 70–79 years, 57.5% of those in their 80 s or older)^31^. In this study, 23.0% of respondents obtained information on cancer prevention through the internet, and the websites of public institutions were associated with a broad range of favorable health behaviors. Given the accessibility of the internet and the movement of a wide range of information sources online (e.g. newspapers, magazines, books, audio and movies), future studies should examine the effectiveness of different online information sources for disseminating information and their effect on cancer prevention. In this study, social media was used by younger generations, and was not associated with health behaviors in this study. HINTS reported that social media represents an effective platform through which the government can communicate health information to the public, particularly populations that may be difficult to reach using traditional forms of media such as younger generations and disadvantaged populations, including those with lower levels of education^32,33^. In terms of education and promotion of HPV vaccination, social media may complement or boost other strategies such as brochures, health classes, and counseling for communicating to younger generations.

The strengths of this study include the nationwide population-based nature of the survey, and the adoption of comparable question items to those of a previous study. However, some limitations warrant mention. First, because this was a cross-sectional study, we could not determine causality in the relationship between information sources and health behaviors. Second, due to the limited cooperation rate, we cannot rule out the possibility of selection bias to reflect characteristics of the respondents. Further, we were unable to examine health information acquisition and interest in disadvantaged populations. Third, analysis was limited due to possible misclassification or unmeasured results owing to the self-reported nature of the questionnaire; unavailable health status information such as medical history and body mass index; and the inability to identify habitual smoking and drinking status due to the limited number of questions. Although we used similar questionnaire items to those in a previous survey, we cannot rule out potential misclassification of information sources that may be related to advances in information and communication technologies. Namely, while traditional media include newspapers, magazines, and books in print form, and television and radio broadcast over radio waves, almost all of these forms of media are now also available online through the internet. Finally, we could not estimate the accuracy of the contents of information sources or the health literacy of participants despite evaluating information sources as a channel for communication in this study.

In conclusion, the Japanese population had greater awareness of smoking and infection as risk factors of cancer, and lower awareness of other lifestyle factors. To effectively inform the public of the significance of cancer prevention, a strategic health communication approach that utilizes various information sources and delivery channels is needed to inform the public about cancer prevention and to motivate risk-reducing behaviors in the population.

## Supplementary Information

Supplementary Information.

## Data Availability

The data are not publicly available due to no approval from the ethics review board.
