# Supplementary material for: A cross-sectional survey on awareness of cancer risk factors, information sources and health behaviors for cancer prevention in Japan
Source: Sci Rep. 2022 Aug 26;12:14606. doi: 10.1038/s41598-022-18853-x (PMC9418251; doi:10.1038/s41598-022-18853-x)
Supplement: Supplementary file 1 — Supplementary Information. [file 41598_2022_18853_MOESM1_ESM.docx]

**Supplementary Information**

**A cross-sectional survey on awareness of cancer risk factors, information sources and health behaviors for cancer prevention in Japan**

Yoko Yamagiwa, Shiori Tanaka, Sarah Krull Abe, Taichi Shimazu, Manami Inoue

**Supplementary Table S1. Survey cooperation rate**

|  | Number of samples | Number of respondents | Cooperation rate (%) ^1)^ |
| --- | --- | --- | --- |
| Total | 4000 | 1216 | 30.4 |
| Region |  |  |  |
| Hokkaido and Tohoku | 462 | 136 | 29.4 |
| Kanto (Kanto and Keihin) | 1354 | 409 | 30.2 |
| Chubu (Koshinetsu, Hokuriku and Tokai) | 726 | 229 | 31.5 |
| Kinki (Kinki and Hanshin) | 648 | 185 | 28.5 |
| Chugoku, Shikoku and Kyusyu | 810 | 257 | 31.7 |
| City-scale of study area |  |  |  |
| Metropolis^2)^ | 1146 | 346 | 30.2 |
| Other city | 2496 | 769 | 30.8 |
| Town and village | 358 | 101 | 28.2 |

^1)^ Number of respondents/number of samples x 100 (%)

^2)^ Sapporo, Sendai, Saitama, Chiba, Tokyo, Yokohama, Kawasaki, Sagami, Niigata, Shizuoka, Hamamatsu, Nagoya, Kyoto, Osaka, Sakai, Kobe, Hiroshima, Okayama, Kitakyushu, Fukuoka, and Kumamoto.

**Supplementary Table S2. Demographic characteristics of respondents who were interested in cancer prevention and who engaged in health behaviors for cancer prevention**

|  | Interest in cancer prevention | *P*-value |  | Engaged in health behaviors for cancer prevention | *P*-value |
| --- | --- | --- | --- | --- | --- |
| Age, mean (95% CI) |  | <0.001^1)^ |  |  | 0.014^1)^ |
| Yes | 56.0 (55.0-57.1) |  |  | 55.8 (54.7-56.8) |  |
| No | 49.8 (47.0-52.5) |  |  | 52.5 (50.1-54.8) |  |
| Sex, yes^3)^ |  | <0.001^2)^ |  |  | 0.0012^2)^ |
| Men | 418 (74.5 + 1.7) |  |  | 372 (67.4 + 2.1) |  |
| Women | 562 (86.3 + 1.3) |  |  | 471 (75.3 + 1.8) |  |
| Educational status, yes^3)^ |  | 0.095^2)^ |  |  | 0.003^2)^ |
| Middle | 68 (74.4 + 4.6) |  |  | 59 (64.0 + 5.4) |  |
| High | 491 (79.7 + 1.5) |  |  | 424 (68.4 + 2.1) |  |
| College or higher | 421 (83.4 + 1.6) |  |  | 390 (77.0 + 1.9) |  |
| City-scale of study area, yes^3)^ |  | 0.319^2)^ |  |  | 0.343^2)^ |
| Metropolis | 278 (80.7 + 2.0) |  |  | 242 (69.7 + 2.9) |  |
| Other city | 616 (80.3 + 1.4) |  |  | 562 (73.2 + 2.0) |  |
| Town and village | 86 (85.4 + 2.5) |  |  | 69 (67.1 + 4.3) |  |

^1)^ Continuous variables were analyzed using weighted data for differences in means.

^2)^ Categorical variables were analyzed using weighted data by the Rao-Scott chi-squared test.

^3)^ Number (% of weighted frequency + standard error)

**Supplementary Table S3. Information source, health behavior, and interest in cancer prevention by age**

| Age | 20-29y | 30-39y | 40-49y | 50-59y | 60-69y | 70-79y | 80y- |
| --- | --- | --- | --- | --- | --- | --- | --- |
|  | (n=118) | (n=179) | (n=200) | (n=197) | (n=223) | (n=197) | (n=102) |
| Interest in cancer prevention, yes^1)^ | 69 (58.4) | 133 (74.1) | 163 (82.3) | 176 (89.3) | 198 (89.0) | 162 (82.6) | 79 (77.5) |
| Health behavior^1)^ |  |  |  |  |  |  |  |
| Improving diet | 24 (20.3) | 48 (27.2) | 75 (37.4) | 89 (45.0) | 101 (45.4) | 86 (43.7) | 40 (38.5) |
| Exercise | 24 (19.6) | 33 (19.0) | 30 (15.0) | 59 (29.9) | 78 (35.0) | 68 (34.3) | 27 (26.3) |
| Cancer screening/health check-up | 16 (13.7) | 33 (18.3) | 75 (37.9) | 88 (45.3) | 100 (44.6) | 81 (41.3) | 22 (21.4) |
| Abstinence from smoking | 36 (30.2) | 77 (42.4) | 73 (36.7) | 75 (38.8) | 87 (39.7) | 60 (31.1) | 30 (29.1) |
| Abstinence from drinking | 21 (17.1) | 39 (21.2) | 35 (17.6) | 40 (20.8) | 50 (23.2) | 43 (22.4) | 25 (24.3) |
| Information source^1)^ |  |  |  |  |  |  |  |
| Television | 85 (72.3) | 137 (76.6) | 165 (82.4) | 151 (76.8) | 185 (83.1) | 176 (89.3) | 87 (85.5) |
| Radio | 3 (2.7) | 7 (4.0) | 10 (5.0) | 26 (13.6) | 26 (11.9) | 26 (12.8) | 13 (12.9) |
| Newspapers | 19 (16.1) | 47 (26.2) | 69 (34.0) | 86 (44.4) | 128 (58.0) | 116 (59.2) | 50 (49.6) |
| Books | 8 (6.9) | 10 (5.5) | 23 (12.1) | 21 (10.4) | 21 (9.5) | 15 (7.5) | 11 (10.4) |
| Magazines | 12 (10.3) | 25 (13.7) | 31 (16.2) | 49 (25.5) | 55 (25.4) | 41 (21.0) | 14 (13.5) |
| Brochures provided by pharmacy/hospital | 20 (16.6) | 30 (17.3) | 33 (16.8) | 47 (24.6) | 49 (21.9) | 38 (19.3) | 13 (12.6) |
| Advertisements | 20 (16.9) | 35 (19.6) | 41 (21.1) | 37 (19.7) | 45 (20.3) | 28 (14.0) | 12 (11.5) |
| Websites of public institution | 13 (11.1) | 29 (16.0) | 30 (15.1) | 27 (14.0) | 24 (10.8) | 14 (7.4) | 4 (3.8) |
| Websites of other organizations | 11 (9.0) | 27 (15.0) | 25 (11.9) | 32 (16.4) | 16 (7.5) | 7 (3.7) | 0 (0) |
| Social networking service | 14 (11.6) | 26 (14.1) | 18 (9.0) | 12 (6.1) | 9 (4.2) | 0 (0) | 1 (1.0) |
| Instructions from professionals | 18 (14.9) | 38 (20.5) | 47 (23.8) | 67 (34.9) | 49 (21.9) | 45 (23.0) | 24 (23.1) |
| Health classes | 7 (5.8) | 6 (3.2) | 15 (7.5) | 28 (14.2) | 23 (10.2) | 21 (10.5) | 8 (7.3) |
| Family/friends | 29 (24.8) | 46 (25.4) | 55 (27.5) | 57 (28.7) | 66 (29.0) | 57 (29.4) | 23 (23.1) |

^1)^ Number (% of weighted frequency)

**Supplementary Table S4. Association between characteristics of users and information sources used by respondents as outcomes on cancer prevention in multivariate-adjusted logistic regression models**

| Information sources | Characteristics of users |  | OR | 95% CI | 99.9231% CI | *P*-value |
| --- | --- | --- | --- | --- | --- | --- |
| Television |  |  |  |  |  |  |
|  | Age | Continuous | 1.01 | 1.00-1.02 | 1.00-1.03 | 0.008 |
|  | Sex | Women vs. Men | 1.95 | 1.49-2.56 | 1.22-3.13 | **<0.0001** |
|  | Education | High vs. Middle | 1.10 | 0.55-2.19 | 0.33-3.65 | 0.7831 |
|  |  | College or higher vs. Middle | 0.98 | 0.47-2.06 | 0.27-3.56 | 0.9616 |
|  | City-scale of study area | Other city vs. Metro | 1.12 | 0.75-1.68 | 0.56-2.27 | 0.5703 |
|  |  | Town vs. Metro | 1.31 | 0.72-2.39 | 0.46-3.74 | 0.3804 |
|  | Interest | Yes vs. Other | 2.34 | 1.60-3.42 | 1.21-4.54 | **<0.0001** |
| Radio |  |  |  |  |  |  |
|  | Age | Continuous | 1.03 | 1.02-1.04 | 1.01-1.05 | **<0.0001** |
|  | Sex | Women vs. Men | 0.83 | 0.57-1.21 | 0.43-1.60 | 0.3243 |
|  | Education | High vs. Middle | 1.30 | 0.65-2.63 | 0.38-4.44 | 0.4562 |
|  |  | College or higher vs. Middle | 1.20 | 0.55-2.63 | 0.31-4.72 | 0.6442 |
|  | City-scale of study area | Other city vs. Metro | 1.38 | 0.83-2.30 | 0.57-3.35 | 0.2134 |
|  |  | Town vs. Metro | 1.16 | 0.65-2.06 | 0.42-3.16 | 0.6168 |
|  | Interest | Yes vs. Other | 1.96 | 1.05-3.67 | 0.66-5.86 | 0.0363 |
| Newspapers | |  |  |  |  |  |
|  | Age | Continuous | 1.04 | 1.03-1.05 | 1.02-1.05 | **<0.0001** |
|  | Sex | Women vs. Men | 0.86 | 0.68-1.08 | 0.57-1.27 | 0.1774 |
|  | Education | High vs. Middle | 1.92 | 1.18-3.14 | 0.82-4.52 | 0.0094 |
|  |  | College or higher vs. Middle | 2.51 | 1.47-4.30 | 0.99-6.40 | 0.0009 |
|  | City-scale of study area | Other city vs. Metro | 1.12 | 0.81-1.55 | 0.63-1.98 | 0.4935 |
|  |  | Town vs. Metro | 1.13 | 0.64-2.02 | 0.41-3.11 | 0.6701 |
|  | Interest | Yes vs. Other | 3.06 | 2.21-4.23 | 1.74-5.39 | **<0.0001** |
| Books |  |  |  |  |  |  |
|  | Age | Continuous | 1.01 | 0.99-1.02 | 0.98-1.03 | 0.2970 |
|  | Sex | Women vs. Men | 1.16 | 0.77-1.75 | 0.57-2.37 | 0.4721 |
|  | Education | High vs. Middle | 9.58 | 1.22-75.0 | 0.27-345.19 | 0.0316 |
|  |  | College or higher vs. Middle | 13.2 | 1.68-103.5 | 0.37-477.7 | 0.0145 |
|  | City-scale of study area | Other city vs. Metro | 1.09 | 0.70-1.70 | 0.50-2.37 | 0.6975 |
|  |  | Town vs. Metro | 1.16 | 0.59-2.27 | 0.36-3.74 | 0.6599 |
|  | Interest | Yes vs. Other | 3.06 | 1.46-6.43 | 0.84-11.16 | 0.0034 |
| Magazines |  |  |  |  |  |  |
|  | Age | Continuous | 1.01 | 1.00-1.02 | 1.00-1.03 | 0.0085 |
|  | Sex | Women vs. Men | 0.94 | 0.70-1.26 | 0.56-1.58 | 0.6653 |
|  | Education | High vs. Middle | 2.69 | 1.24-5.81 | 0.70-10.29 | 0.0123 |
|  |  | College or higher vs. Middle | 3.58 | 1.57-8.20 | 0.85-15.15 | 0.0028 |
|  | City-scale of study area | Other city vs. Metro | 1.00 | 0.68-1.44 | 0.51-1.91 | 0.9527 |
|  |  | Town vs. Metro | 1.53 | 0.82-2.85 | 0.51-4.53 | 0.1838 |
|  | Interest | Yes vs. Other | 4.12 | 2.36-7.17 | 1.56-10.83 | **<0.0001** |
| Brochures provided by pharmacy/hospital | | |  |  |  |  |
|  | Age | Continuous | 1.00 | 0.99-1.01 | 0.99-1.02 | 0.6590 |
|  | Sex | Women vs. Men | 1.31 | 0.99-1.75 | 0.80-2.16 | 0.0630 |
|  | Education | High vs. Middle | 2.57 | 1.13-5.84 | 0.62-10.75 | 0.0246 |
|  |  | College or higher vs. Middle | 2.80 | 1.25-6.24 | 0.69-11.32 | 0.0125 |
|  | City-scale of study area | Other city vs. Metro | 1.04 | 0.71-1.53 | 0.54-2.03 | 0.8302 |
|  |  | Town vs. Metro | 0.73 | 0.41-1.29 | 0.27-1.98 | 0.2770 |
|  | Interest | Yes vs. Other | 2.37 | 1.48-3.78 | 1.05-5.35 | **0.0004** |
| Advertisements | |  |  |  |  |  |
|  | Age | Continuous | 0.99 | 0.98-1.00 | 0.98-1.01 | 0.0504 |
|  | Sex | Women vs. Men | 0.85 | 0.64-1.14 | 0.51-1.42 | 0.2795 |
|  | Education | High vs. Middle | 1.08 | 0.59-1.98 | 0.37-3.11 | 0.8095 |
|  |  | College or higher vs. Middle | 1.08 | 0.55-2.11 | 0.34-3.47 | 0.8166 |
|  | City-scale of study area | Other city vs. Metro | 1.36 | 0.87-2.11 | 0.63-2.93 | 0.1741 |
|  |  | Town vs. Metro | 1.42 | 0.79-2.56 | 0.51-3.96 | 0.2456 |
|  | Interest | Yes vs. Other | 2.87 | 1.73-4.75 | 1.19-6.90 | **<0.0001** |
| Websites of public institution | |  |  |  |  |  |
|  | Age | Continuous | 0.99 | 0.97-1.00 | 0.97-1.00 | 0.0046 |
|  | Sex | Women vs. Men | 0.89 | 0.62-1.27 | 0.47-1.65 | 0.4996 |
|  | Education | High vs. Middle | 3.63 | 0.88-15.0 | 0.31-42.95 | 0.0744 |
|  |  | College or higher vs. Middle | 5.89 | 1.40-24.8 | 0.48-72.02 | 0.0160 |
|  | City-scale of study area | Other city vs. Metro | 0.82 | 0.53-1.28 | 0.38-1.77 | 0.3782 |
|  |  | Town vs. Metro | 0.74 | 0.32-1.71 | 0.18-3.16 | 0.4820 |
|  | Interest | Yes vs. Other | 3.84 | 1.98-7.45 | 1.21-12.18 | **0.0001** |
| Websites of other organizations | |  |  |  |  |  |
|  | Age | Continuous | 0.98 | 0.97-0.99 | 0.96-1.00 | **0.0007** |
|  | Sex | Women vs. Men | 0.59 | 0.39-0.90 | 0.28-1.24 | 0.0155 |
|  | Education | High vs. Middle | 5.06 | 0.71-36.3 | 0.16-156.38 | 0.1058 |
|  |  | College or higher vs. Middle | 9.09 | 1.22-67.8 | 0.27-301.46 | 0.0316 |
|  | City-scale of study area | Other city vs. Metro | 0.64 | 0.42-0.99 | 0.30-1.36 | 0.0442 |
|  |  | Town vs. Metro | 0.56 | 0.24-1.31 | 0.13-2.46 | 0.1826 |
|  | Interest | Yes vs. Other | 2.09 | 1.21-3.60 | 0.81-5.38 | 0.0083 |
| Social networking service | |  |  |  |  |  |
|  | Age | Continuous | 0.96 | 0.94-0.97 | 0.93-0.98 | **<0.0001** |
|  | Sex | Women vs. Men | 1.18 | 0.74-1.87 | 0.52-2.65 | 0.4955 |
|  | Education | High vs. Middle | 0.67 | 0.24-1.90 | 0.11-4.08 | 0.4508 |
|  |  | College or higher vs. Middle | 0.88 | 0.29-2.67 | 0.13-6.09 | 0.8173 |
|  | City-scale of study area | Other city vs. Metro | 0.76 | 0.41-1.39 | 0.27-2.17 | 0.3655 |
|  |  | Town vs. Metro | 1.29 | 0.43-3.84 | 0.19-8.66 | 0.6500 |
|  | Interest | Yes vs. Other | 1.91 | 0.92-3.99 | 0.53-6.89 | 0.0844 |
| Instructions from professionals | |  |  |  |  |  |
|  | Age | Continuous | 1.00 | 0.99-1.01 | 0.99-1.02 | 0.7656 |
|  | Sex | Women vs. Men | 0.99 | 0.77-1.27 | 0.64-1.52 | 0.9244 |
|  | Education | High vs. Middle | 1.10 | 0.61-1.96 | 0.40-3.03 | 0.7562 |
|  |  | College or higher vs. Middle | 1.14 | 0.60-2.19 | 0.37-3.54 | 0.6906 |
|  | City-scale of study area | Other city vs. Metro | 1.13 | 0.77-1.65 | 0.58-2.19 | 0.5439 |
|  |  | Town vs. Metro | 0.89 | 0.50-1.61 | 0.32-2.49 | 0.7075 |
|  | Interest | Yes vs. Other | 3.42 | 2.02-5.84 | 1.36-8.66 | **<0.0001** |
| Health classes | |  |  |  |  |  |
|  | Age | Continuous | 1.02 | 1.00-1.03 | 1.00-1.04 | 0.0087 |
|  | Sex | Women vs. Men | 2.44 | 1.62-3.68 | 1.19-5.00 | **<0.0001** |
|  | Education | High vs. Middle | 1.98 | 0.67-5.81 | 0.30-12.94 | 0.2136 |
|  |  | College or higher vs. Middle | 3.32 | 1.06-10.3 | 0.46-24.03 | 0.0390 |
|  | City-scale of study area | Other city vs. Metro | 0.96 | 0.56-1.64 | 0.37-2.46 | 0.8671 |
|  |  | Town vs. Metro | 1.82 | 0.86-3.85 | 0.49-6.71 | 0.1159 |
|  | Interest | Yes vs. Other | 2.12 | 1.07-4.20 | 0.64-6.97 | 0.0316 |
| Family/friends | |  |  |  |  |  |
|  | Age | Continuous | 1.00 | 0.99-1.01 | 0.99-1.01 | 0.8110 |
|  | Sex | Women vs. Men | 1.76 | 1.37-2.25 | 1.14-2.70 | **<0.0001** |
|  | Education | High vs. Middle | 2.03 | 1.10-3.75 | 0.70-5.91 | 0.0235 |
|  |  | College or higher vs. Middle | 1.41 | 0.73-2.72 | 0.45-4.42 | 0.3060 |
|  | City-scale of study area | Other city vs. Metro | 0.64 | 0.44-0.92 | 0.34-1.21 | 0.0169 |
|  |  | Town vs. Metro | 0.75 | 0.39-1.42 | 0.24-2.30 | 0.3685 |
|  | Interest | Yes vs. Other | 2.89 | 1.88-4.42 | 1.37-6.07 | **<0.0001** |

Associations between users’ characteristics and information sources were analyzed by assigning 5 users’ characteristics (age, sex, educational status, city-scale of study area, and interest in cancer prevention) as explanatory variables and 13 individual information sources as outcomes in logistic regression models. The significance level was 0.00077 by Bonferroni correction for multiple comparisons. The significance level was 0.00077 by Bonferroni correction for multiple comparisons and 99.9231% CI was calculated based on Bonferroni correction.

OR: odds ratio; CI: confidence interval.

**Supplementary file 1. Structured questionnaire sheet for face-to-face interview**

Central Research Services, Inc.

December 2018

- (Survey on Daily Life) December ○

| Branch office | | Location | | | Target | |
| --- | --- | --- | --- | --- | --- | --- |
|  |  |  |  |  |  |  |

F1. (Occupation) Please tell me about your occupation. [Investigator memo: Start time HH:MM] Sheet No.= 01

| 1 | 2 | 3 | 4 | 5 | 6 | 7 | 8 |
| --- | --- | --- | --- | --- | --- | --- | --- |
| Agriculture, forestry, fishery including family employee | Commerce and industry, service industry including family employee | Clerical work | Labour work | Freelance work/ Management work | Full-time homemaker | Student | Others/No occupation |

F2. (Sex)

| 1 | 2 |
| --- | --- |
| Male | Female |

F3. (Age)

years old

F4. (Education) Please tell me about your final academic level.

| 1 | 2 | 3 |
| --- | --- | --- |
| (New system) Middle school | (New system) High school | (New system) Junior college, university |
| (Old system) Elementary school, higher elementary school | (Old system) Middle school | (Old system) High school, technical college, university |

Next, we would like to ask about “cancer”

Q1. [Answer Sheet 1] What percentage do you think each of the following items accounts for as part of the overall cause of cancer?

Please answer for each item a) to m).

1. How about drinking?

[Investigator note: Similarly, ask on b) to m)]

|  | (i) | (ii) | (iii) | (iv) | (v) | (vi) | (vii) | (viii) | (ix) | (x) | (xi) | (xii) | (xiii) |  |
| --- | --- | --- | --- | --- | --- | --- | --- | --- | --- | --- | --- | --- | --- | --- |
|  | >5% | 5 to <10% | 10 to <15% | 15 to <20% | 20 to <25% | 25 to <30% | 30 to <40% | 40 to <50% | 50 to <60% | 60 to <70% | 70 to <80% | 80 to <90% | 90 to 100% | I don’t know |
| a) “Drinking” | 1 | 2 | 3 | 4 | 5 | 6 | 7 | 8 | 9 | 10 | 11 | 12 | 13 | 14 |
| b) “Unbalanced diet such as lack of vegetables/  fruits and/or excessive salt” | 1 | 2 | 3 | 4 | 5 | 6 | 7 | 8 | 9 | 10 | 11 | 12 | 13 | 14 |
| c) “Food additives/pesticide residues” | 1 | 2 | 3 | 4 | 5 | 6 | 7 | 8 | 9 | 10 | 11 | 12 | 13 | 14 |
| d) “Burnt fish and meat” | 1 | 2 | 3 | 4 | 5 | 6 | 7 | 8 | 9 | 10 | 11 | 12 | 13 | 14 |
| e) “Smoking” | 1 | 2 | 3 | 4 | 5 | 6 | 7 | 8 | 9 | 10 | 11 | 12 | 13 | 14 |
| f) “Obesity” | 1 | 2 | 3 | 4 | 5 | 6 | 7 | 8 | 9 | 10 | 11 | 12 | 13 | 14 |
| g) “Lack of exercise” | 1 | 2 | 3 | 4 | 5 | 6 | 7 | 8 | 9 | 10 | 11 | 12 | 13 | 14 |
| h) “Dioxins and other endocrine disrupting  chemicals (environmental hormones)” | 1 | 2 | 3 | 4 | 5 | 6 | 7 | 8 | 9 | 10 | 11 | 12 | 13 | 14 |
| i) “Air pollution such as diesel exhaust” | 1 | 2 | 3 | 4 | 5 | 6 | 7 | 8 | 9 | 10 | 11 | 12 | 13 | 14 |
| j) “Hazardous substance exposure at workplace” | 1 | 2 | 3 | 4 | 5 | 6 | 7 | 8 | 9 | 10 | 11 | 12 | 13 | 14 |
| k) “Bacteria and viruses that cause cancer” | 1 | 2 | 3 | 4 | 5 | 6 | 7 | 8 | 9 | 10 | 11 | 12 | 13 | 14 |
| l) “Stress” | 1 | 2 | 3 | 4 | 5 | 6 | 7 | 8 | 9 | 10 | 11 | 12 | 13 | 14 |
| m) “Genes” | 1 | 2 | 3 | 4 | 5 | 6 | 7 | 8 | 9 | 10 | 11 | 12 | 13 | 14 |

Q2. By what percentage do you think it is possible to decrease the possibility of developing cancer by improving lifestyle? Please answer as a percentage. If you think the possibility of developing cancer would decline to 3/4 of the present rate, this would be a 25% decline; if to half the present rate, this would be a 50% decline; and if to 1/4 the present rate, this would be a 75% decline.

% decline

Q3. [Answer Table 2] How interested are you in “cancer prevention”? Please select one from below.

| 1 | 2 | 3 | 4 | 5 |
| --- | --- | --- | --- | --- |
| (a) | (b) | (c) | (d) |  |
| Very interested | Somewhat interested | Not so interested | Not interested at all | Don’t know |

Q4. [Answer Table 3] Where do you obtain information on “cancer prevention”? Select as many items as you wish from below. (M.A.)

| 1 | (a) | Television | 11 | (k) | Direct mail |
| --- | --- | --- | --- | --- | --- |
| 2 | (b) | Radio | 12 | (l) | Instructions from professionals such as doctors and nurses |
| 3 | (c) | Newspaper articles | 13 | (m) | Health classes/lectures |
| 4 | (d) | Magazine articles | 14 | (n) | Friends and acquaintances |
| 5 | (e) | Newspaper/magazine advertisements | 15 | (o) | Websites of public institutions |
| 6 | (f) | Insert advertisements | 16 | (p) | Other websites |
| 7 | (g) | Related technical books/books | 17 | (q) | SNS; Facebook, Twitter, etc. |
| 8 | (h) | Posters inside trains, stations/outdoor advertisements | 18 | (r) | Others ( ) |
| 9 | (i) | Pharmacy store signboards/posters | 19 |  | Nowhere in particular |
| 10 | (j) | Pharmacy/hospital handouts | 20 |  | Don’t know |

Q5. [Answer Table 4] Is there anything that you do regularly to consciously prevent cancer? Select all items that apply to this from below. (M.A.)

| 1 | (a) | Improve my diet | 6 | (f) | Refrain from smoking, reduce number of cigarettes |
| --- | --- | --- | --- | --- | --- |
| 2 | (b) | Take health foods/supplements | 7 | (g) | Refrain from drinking, reduce drinking amount |
| 3 | (c) | Exercise | 8 | (h) | Others ( ) |
| 4 | (d) | Relieve stress through hobbies, etc. | 9 |  | Nothing in particular |
| 5 | (e) | Take cancer screening/general medical checkups | 10 |  | Don’t know |

Q6. [Answer Table 5] Suppose that there was a test that would examine your genes to see whether you had a physical constitution which made you susceptible to developing cancer.

1. If you could avoid developing cancer by improving your lifestyle, would you consider taking the test?

| 1 | 2 | 3 | 4 | 5 |
| --- | --- | --- | --- | --- |
| (a) | (b) | (c) | (d) |  |
| Strongly agree | Somewhat agree | Somewhat disagree | Strongly disagree | Don’t know |

1. If you could not avoid developing cancer even by prevention measures, such as improving your lifestyle, would you consider taking the test?

| 1 | 2 | 3 | 4 | 5 |
| --- | --- | --- | --- | --- |
| (a) | (b) | (c) | (d) |  |
| Strongly agree | Somewhat agree | Somewhat disagree | Strongly disagree | Don’t know |

Q7. Have you ever taken a genetic test that examines whether you have a physical constitution which makes you susceptible to developing cancer?

| 1 | 2 | 3 |
| --- | --- | --- |
| Yes | No | I don’t know |

(continued on next page)

SQ. [Answer Table 6] What did you think about the results of the genetic test? Select one answer each for of a) to c).

1. Do you think “it is reliable”?

[Investigator note: Ask (b) and (c) similarly]

|  |  | (i) | (ii) | (iii) | (iv) | (v) |  |
| --- | --- | --- | --- | --- | --- | --- | --- |
|  |  | Strongly agree | Somewhat agree | Neither agree nor disagree | Somewhat disagree | Strongly disagree | Don’t know |
| a) | “It is reliable” | 1 | 2 | 3 | 4 | 5 | 6 |
| b) | “I can understand the meaning of the result” | 1 | 2 | 3 | 4 | 5 | 6 |
| c) | “It can be used to improve lifestyle to prevent cancer” | 1 | 2 | 3 | 4 | 5 | 6 |
